# Hematological features in children with systemic lupus erythematosus: are they more common than appreciated?

**DOI:** 10.1186/1546-0096-9-S1-P242

**Published:** 2011-09-14

**Authors:** Muge Gokce, Nesrin Besbas, Yelda Bilginer, Mualla Cetin, Fatma Gumruk, Seza Ozen

**Affiliations:** 1 Hacettepe Medical Faculty, Pediatric Hematology Division, Ankara, Turkey; 2 Hacettepe Medical Faculty, Pediatric Rheumatology Division, Ankara, Turkey

## Aim

Hematological involvement is an important element of morbidity in systemic lupus erythematosus (SLE). In this retrospective study, we describe the hematological features that were observed in children with SLE in our pediatric center.

## Methods

We evaluated the hematological findings of 40 children (33 female) with SLE diagnosed and followed at the Pediatric Rheumatology Division of Hacettepe University, Turkey.

## Results

Median age at presentation was 12 years. The most common hematological finding was anemia (n=27). Of these, Coombs test was positive in nineteen (70.3%). Leukopenia, neutropenia and thrombocytopenia were detected in 32.5% (n=13), 17.5 % (n=7) and 32.5% (n=13), respectively.

Bone marrow aspiration was performed in fifteen (37.5%) mainly to assess the pancytopenia. Secondary dysplastic changes in myeloid and erythroid lineage, increased histiocytes and erythroid lineage hyperactivity were common findings by bone marrow evaluation. Acute lymphoblastic leukemia was diagnosed in a girl nine months after an initial diagnosis of SLE. She had received corticosteroids and azothiopurine for SLE.

Evidence of hemophagocytosis was present in bone marrow smears of five patients, one of these patients had a resistant course of severe macrophage activating syndrome and was treated with HLH 2004 protocole and plasmapheresis. She died of secondary infections and multiorgan failure.

Antiphospholipid and anticardiolipin antibodies were positive in 11 and 13 of the patients, retrospectively. Of these patients, four developed deep vein thrombosis, one cerebral sinus thrombosis and one presented with purpura fulminans.

## Conclusion

Hematological should be carefully assessed and treated vigorously to prevent the morbidity and possible mortality.

